# Association of prognostic nutritional index with mortalities in American adult cancer survivors: A cohort study based on NHANES, 1999–2018

**DOI:** 10.1002/fsn3.3877

**Published:** 2023-11-30

**Authors:** Li Zhao, Xia Shen, Long Yang, Pengfei Wang, Jianfeng Zhang, Ning Liu, Yan Xie

**Affiliations:** ^1^ Department of Nursing, Wuxi Maternity and Child Health Care Hospital, Women's Hospital of Jiangnan University Jiangnan University Wuxi Jiangsu China; ^2^ Department of Nursing, Wuxi Medical College Jiangnan University Wuxi Jiangsu China; ^3^ College of Pediatrics Xinjiang Medical University Urumqi China; ^4^ Department of Anorectal Surgery China Academy of Chinese Medical Sciences Xi Yuan Hospital Beijing China; ^5^ Department of the Office of Science and Education, Wuxi Maternity and Child Health Care Hospital, Women's Hospital of Jiangnan University Jiangnan University Wuxi Jiangsu China; ^6^ Department of Hospital Sentinel Medicine, Wuxi Maternity and Child Health Care Hospital, Women's Hospital of Jiangnan University Jiangnan University Wuxi Jiangsu China

**Keywords:** adult, all‐cause mortality, cancer survivors, cardiovascular mortality, malignant tumor mortality, prognostic nutritional index

## Abstract

The prognostic nutritional index (PNI) has been associated with disease progression and overall survival among cancer patients. Nonetheless, the association between PNI and mortality risk in adult cancer patients within the United States remains unexplored. This study aims to elucidate the connection between PNI and prognostic outcomes in American adult cancer patients. This cohort study derived data from the National Health and Nutrition Examination database, involving 4366 American adults diagnosed with cancer between 1999 and 2018. The nutritional status was assessed using the PNI, with higher PNI scores indicating a more favorable nutritional status. The study employed Kaplan–Meier curves and Cox proportional hazard regression to investigate the impact of PNI on various outcomes, including all‐cause mortality (ACM), cardiovascular mortality (CAM), and malignancy tumor mortality (MTM) among adult cancer patients. Furthermore, restricted cubic spline models were used to examine the potential nonlinear relationship between the variables by creating hazard ratio (HR) curves at four specific points. The median follow‐up duration was 84 months, during which 1530 (35.04%) cases of ACM occurred, including 331 (13.67%) CAM and 449 (10.45%) MTM. COX regression analysis revealed a significant inverse association between PNI and patient prognosis, with HRs of 0.95 (95% CI: 0.93–0.96, *p* < .001) for ACM, 0.93 (95% CI: 0.90–0.96, *p* < .001) for CAM, and 0.94 (95% CI: 0.91–0.97, *p* < .001) for MTM. Both Kaplan–Meier analyses and restricted cubic spline curves showed significant differences in mortality rates related to PNI (*p* < .001, nonlinear *p* < .001). Our study provides compelling evidence of a clear association between PNI and reduced risk of ACM, CAM, and MTM in adult cancer patients in the United States. These findings underscore the significance of incorporating PNI as a possible prognostic indicator for individuals diagnosed with cancer.

## Graphical abstract

Prognostic nutritional index (PNI) is associated with a reduced risk of all‐cause mortality, cardiovascular mortality, and death from malignant tumors in adult cancer patients in America. The findings highlight the importance of using PNI as a potential prognostic indicator for cancer patients.

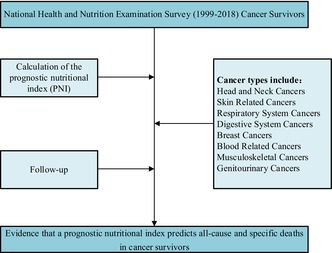

## 1 INTRODUCTION

Cancer is a widespread and serious global health issue, representing one of the common causes of mortality and posing significant challenges to patients' quality of survival and prognosis. Predictions indicate that the United States could witness a staggering surge in newly diagnosed cancer cases by the year 2030, potentially reaching an alarming 22.1 million cases (Miller et al., 2019). Cancer often imposes tremendous financial stress and psychological burden on patients, their families, and society.

Nutrition serves as the foundation for maintaining normal physiological function and immunity in the body. However, both cancer itself and anticancer treatments can profoundly impact the state of nutrition. Consequently, cancer patients often experience heightened metabolic demands, increased energy expenditure, loss of appetite, and inadequate nutritional intake, all of which can impact their prognosis. Up to now, several nutritional and immunological indices have been identified to predict the prognosis of cancer patients, among which the prognostic nutrition index (PNI), originally proposed by Buzby et al. (1980), has been widely utilized due to its simplicity and convenience in assessment. PNI is a critical indicator used to comprehensively evaluate patients' nutritional and metabolic status, calculated based on the amalgamation of serum albumin and peripheral blood lymphocyte counts.

In recent years, many studies (Ellez et al., 2023; Mohri et al., 2016; Mori et al., 2015; Nogueiro et al., 2022; Tokunaga et al., 2015; Zheng et al., 2022) have demonstrated that a lower PNI is an independent predictor of long‐term poorer prognosis across various cancer types, including gastric, breast, non‐small‐cell lung, colorectal, and esophageal cancers. However, the sample sizes of these studies were small enough that the conclusions warrant further validation. Further, to our knowledge, no research has yet evaluated the relationship between PNI and prognosis in adult cancer population in America. Given the high incidence of cancer in the United States, it is critical to evaluate whether PNI can predict the prognosis of patients with cancer. To bridge this knowledge gap, we intend to prospectively investigate the association of PNI with the risk of all‐cause mortality (ACM), cardiovascular mortality (CAM), and malignancy tumor mortality (MTM) in a large, nationally representative sample of participants with cancer in the United States. This aims to provide additional health‐guidance strategies to improve the survival of US cancer patients and ultimately improve public health.

## 2 METHODS

### 2.1 Study participants

The data for this study were sourced from the National Health and Nutrition Examination Survey (NHANES), which spanned 10 consecutive cycles from 1999 to 2018. NHANES was administered by the National Center for Health Statistics, under the auspices of the US Centers for Disease Control and Prevention. This nationally representative cohort‐sectional survey utilized a grouped probability design and intricate stratified sampling methodology, targeting noninstitutionalized US civilians. The survey program encompassed demographic surveys, physical examinations, laboratory indicator tests, dietary profiles, and psychophysiological questionnaire assessments, which could be used to evaluate the relationship between nutritional status and disease avoidance.

**FIGURE 1:**
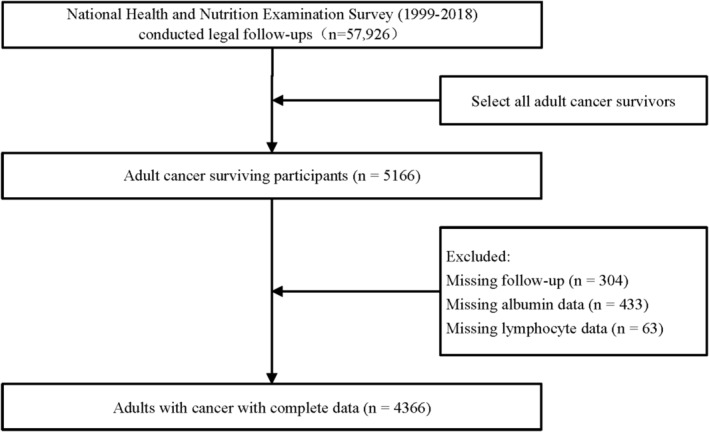
Flowchart depicting study participant inclusion and exclusion.

The study strictly adhered to the guidelines of STROBE (von Elm et al., 2007). Among 57,926 participants in the 1999–2018 cycle of the NHANES survey, legal follow‐ups were conducted, from which we selected cancer survivors older than 18 years (*N* = 5166). Furthermore, participants with missing data were excluded from the analysis, including missing follow‐up (*N* = 304 people), missing albumin data (*N* = 433), and missing blood lymphocyte data (*N* = 63). As shown in Figure 1, a final total of 4366 samples were left in the analysis. The data were analyzed during the period from June 2023 through July 2023. NHANES obtained ethical approval from the Ethics Review Committee of the National Center for Health Statistics, with the following ethics approval numbers: Protocol #2018‐01, Continuation of Protocol #2011‐17, Protocol #2011‐17, Continuation of Protocol #2005‐06, Protocol #2005‐06, and Protocol #98‐12. Given that this study involved a secondary analysis of NHANES data, informed consent from patients was not required. Additional information can be obtained from the following website: NCHS Ethics Review Board Approval (cdc.gov). The study data are accessible on the website: https://www.cdc.gov/nchs/nhanes/index.htm.

### 2.2 Assessment of prognostic nutritional index

The formula for calculating PNI: 5 × lymphocyte count (10^9^/L) + serum albumin (g/L). Participants were allowed to sit still for at least 15 min prior to specimen collection. After blood specimens were collected, the specimens were mixed well to allow for complete clot formation and centrifuged at 2000×*g* for 10 min before use. The complete blood count parameters were determined according to the Beckman Coulter® counting and quantification method to obtain the corresponding lymphocyte count (NHANES, 2006). Serum albumin count was performed using a Roche Cobas 6000 analyzer (Roche Diagnostics Corporation, Indianapolis, IN 46250) with bromocresol purple reagent (Prevention).

### 2.3 Outcome assessment

Study outcomes included ACM, CAM, and MTM, each considered separately. ACM were considered as the probability of deaths from any cause, while CAM were identified as the probability of deaths originating from a variety of cardiovascular diseases (CVDs) including heart disease (e.g., myocardial infarction and sudden cardiac death) as well as cerebrovascular disease (stroke) (Wen et al., 2023). MTM were viewed as the probability of deaths due to a variety of malignant tumors, including head and neck cancers, skin‐related cancers, respiratory system cancers, digestive system cancers, breast cancers, blood‐related cancers, musculoskeletal cancers, genitourinary cancers, and other cancers. Mortality data for the follow‐up participants were derived from the NHANES Public Use Linked Mortality File. Within this dataset, the National Center for Health Statistics established a correlation with the National Death Index using a probabilistic algorithm for matching. In this case, data on cardiovascular deaths are taken from the International Statistical Classification of Diseases, 10th edition (ICD‐10) codes including I20–I51, I13, I11, and I00–I09 (Shen et al., 2023). Every patient's follow‐up is extended until the occurrence of their passing or until the study's conclusion (Bottomley & Raymond, 2007). Every patient's follow‐up is extended until the occurrence of their passing or until the study's conclusion (December 31, 2019).

### 2.4 Selection of study variables

The inclusion of covariates in this study is rooted in a comprehensive review of previous literature, clinical expertise, and the attainment of statistical significance (Liu et al., 2022; Qiao et al., 2023; Ying et al., 2022). During home interviews, demographic characteristics such as age, gender, race/ethnicity, marital status, and education levels were collected in the study. Physical measurements were taken by trained technicians and included height, weight, and the mean of three consecutive blood pressure readings obtained while the participants were sitting and at rest for 5 min.

Lifestyle factors, including smoking, alcohol consumption, and physical activity, were captured through self‐reported responses. Lifestyle factors, such as smoking, alcohol consumption, and physical activity, were obtained by self‐report. “Never” were identified as those who had smoked <100 cigarettes in their lifetime; those who had smoked ≥100 cigarettes in their lifetime and were now nonsmokers were considered “former”; those who had smoked ≥100 cigarettes in their lifetime and had smoked every day were categorized as “Now.” Participants were regarded as “never,” “former,” “mild,” and “moderate or heavy” drinkers according to the number of drinks he/she had drunk every day in 1 year: “Never” (had <12 drinks in lifetime), “former” (did not drink last year and ≥12 drinks/year or did not drink last year but drank ≥12 drinks in lifetime), “mild” (women drink less than 2 drinks per day and men drink less than 4 drinks per day), and “moderate or heavy” (women consume at least 2 drinks per day and men consume at least 4 drinks per day). Physical activity is categorized as “poor,” “intermediate,” and “ideal” according to metabolic equivalent task (MET) score. Biochemical data such as Lymphocytes, Creatinine (Cr), Neutrophils, triglyceride (TG), uric acid (UA), total cholesterol (TC), and albumin were obtained from the file of blood Hemal Biochemistry. Additionally, the energy intake was assessed using dietary data obtained from 24‐hour dietary recalls.

Participants with systolic blood pressure values (mmHg) ≥140 and/or diastolic blood pressure values (mmHg) ≥90 or individuals with a reported history of high blood pressure and a physician's diagnosis of having hypertension were defined as hypertensive. The diagnostic for diabetes includes these conditions, one of which should be met: doctor's notification of diabetes mellitus, fasting blood glucose ≥7.0 (mmol/L), glycosylated hemoglobin >6.5 (%), random blood glucose ≥11.1 (mmol/L), two‐hour OGTT blood glucose ≥11.1 (mmol/L), and use of antihyperglycemic drugs or insulin. CVD was viewed as having been diagnosed by a doctor as having a coronary heart disease/stroke/heart attack. In addition, chronic kidney disease (CKD) is defined as an estimated glomerular filtration rate <60 mL/min/1.73 m^2^.

### 2.5 Statistical analysis

Statistical analyses were carried out using the R software (The R Foundation version 4.2.3). A two‐sided *p*‐value lower than .05 was deemed to be statistically significant. To ensure the accuracy of results, the data were weighted in accordance with the NHANES analytical guidelines. This weighting aimed to accommodate the intricate sampling design inherent to this dataset (Johnson et al., 2014). Categorical variables were presented as weighted frequencies and corresponding percentages. To assess group differences, Rao‐Scott's *χ* ^2^ test was employed. Continuous variables were described as weighted means ± standard error. Group comparisons for continuous variables were conducted using one‐way analysis of variance.

Cox proportional hazard regression modeling was employed to examine the association of PNI with mortality among cancer survivors. The crude model did not involve any adjustment. Model 1 adjusted for a range of characteristics, including gender, age, marital status, race/ethnicity, body mass index, smoking status, and alcohol consumption. Model 2 adds neutrophil count, Cr, UA, TG, and TC to Model 1. In Model 3, Adjustments for diabetes, hypertension, CKD, and CVD were added to Model 2. Further, to explore the continuum relationship between PNI and three mortality outcomes, a weight‐multivariate adjusted Cox‐constrained cubic spline model was formulated. This was constructed at the 5th, 35th, 65th, and 95th percentile of the PNI. Additionally, the PNI was stratified into four quartiles: Q1 (<48.0), Q2 (48.0 to 51.0), Q3 (51.1 to 54.5), and Q4 (>54.5).

In addition, Kaplan–Meier curves with log‐rank tests were used to evaluate the survival probability of cancer patients across different quartile levels of PNI. Finally, subgroup analyses were performed on different age groups, genders, and cancer categories to verify the stability of the relationship between PNI and the prognosis of cancer patients.

### 2.6 Participants and public involvement

Participants were not involved in the design and dissemination plan of our study.

## 3 RESULTS

### 3.1 Baseline characteristics

Data were extracted from NHANES for 10 cycles spanning from 1999–2000 through 2017–2018. This cohort study included a total of 4366 participants, representing an estimated population of 18,733,193 individuals based on weighting. Baseline characteristics of these 4366 participants according to PNI quartiles are presented in Table 1. The PNI scores ranged from 51.57 to 51.79 in the sample, with a mean PNI score of 45.38 ± 0.08 (mean ± SD) for participants in quartile 1, and 57.95 ± 0.09 for participants in quartile 4. The average age of the participants was 52.31 years, with the majority being male (2300, 52.7%) and 3075 (70.4%) being non‐Hispanic white. In quartile 4, participants had higher neutrophil and lymphocyte counts, lower Cr levels, higher TG, TC, and albumin levels, and were more likely to have never smoked, engage in intermediate‐intensity physical activity, be light drinkers, have no CVD, and have no hypertension.

**TABLE 1:** Baseline information for participants grouped according to PNI, weight.

Characteristics	Overall	Q1 (<48.0)	Q2 (48.0 to 51.0)	Q3 (51.1 to 54.5)	Q4 (>54.5)	*p*‐value
	*N* = 4366	*N* = 1183	*N* = 1102	*N* = 1102	*N* = 979	
PNI	51.68 ± 0.11	45.38 ± 0.08	49.81 ± 0.03	52.97 ± 0.04	57.95 ± 0.09	**<.001**
Age, year	62.31 ± 0.31	68.26 ± 0.57	63.87 ± 0.48	60.56 ± 0.52	57.17 ± 0.64	**<.001**
Gender						
Male	2300 (52.7)	573 (55.1)	564 (58.3)	598 (57.9)	565 (59.2)	.517
Female	2066 (47.3)	610 (44.9)	538 (41.7)	504 (42.1)	414 (40.8)	
Race						
Non‐Hispanic White	3075 (70.4)	817 (84.9)	811 (88.5)	775 (86.5)	672 (87.0)	**.017**
Non‐Hispanic Black	567 (13)	199 (7.1)	125 (4.3)	134 (4.8)	109 (4.2)	
Mexican American	317 (7.3)	69 (2.1)	73 (2.0)	81 (2.3)	94 (3.1)	
Other race	407 (9.3)	98 (5.8)	93 (5.3)	112 (6.5)	104 (5.7)	
Marital						
With‐partner	2650 (60.7)	658 (61.3)	699 (67.9)	669 (65.3)	624 (68.2)	**<.001**
Without‐partner	1435 (32.9)	466 (35.2)	338 (25.6)	352 (28.5)	279 (23.6)	
Never married	281 (6.4)	59 (3.5)	65 (6.5)	81 (6.2)	76 (8.2)	
Education						
High school or above	3910 (89.6)	1063 (94.3)	980 (95.1)	988 (95.0)	879 (94.5)	.793
Less than high school	456 (10.4)	120 (5.7)	122 (4.9)	114 (5.0)	100 (5.5)	
Height, cm	167.54 ± 0.22	167.07 ± 0.40	167.50 ± 0.38	167.67 ± 0.37	167.86 ± 0.44	.530
Weight, kg	80.88 ± 0.37	81.44 ± 0.81	81.66 ± 0.85	80.89 ± 0.70	79.55 ± 0.79	.228
BMI, kg/m^2^	28.72 ± 0.12	29.10 ± 0.25	28.99 ± 0.28	28.69 ± 0.23	28.16 ± 0.24	**.030**
MET						
Poor	1571 (36)	431 (34.9)	400 (32.3)	389 (33.5)	351 (35.9)	.533
Intermediate	2454 (56.2)	669 (58.9)	622 (58.9)	622 (58.1)	541 (56.4)	
Ideal	341 (7.8)	83 (6.2)	80 (8.9)	91 (8.4)	87 (7.7)	
Drinking						
Never	940 (21.5)	317 (24.9)	233 (17.3)	211 (15.5)	179 (14.6)	**<.001**
Former	977 (22.4)	286 (21.8)	251 (19.0)	241 (17.5)	199 (16.2)	
Mild	1648 (37.7)	439 (39.9)	420 (41.4)	422 (41.6)	367 (38.6)	
Moderate or heavy	801 (18.3)	141 (13.4)	198 (22.3)	228 (25.3)	234 (30.6)	
Smoking						
Never	1942 (44.5)	521 (47.3)	502 (46.6)	524 (49.4)	395 (38.4)	**<.001**
Former	1741 (39.9)	523 (41.9)	482 (41.7)	400 (33.9)	336 (34.4)	
Now	679 (15.6)	137 (10.8)	118 (11.7)	178 (16.6)	246 (27.1)	
Energy intake						
Low	2183 (50)	596 (46.2)	528 (43.7)	567 (48.1)	492 (47.0)	.403
High	2183 (50)	587 (53.8)	574 (56.3)	535 (51.9)	487 (53.0)	
Biochemical indicators						
Neutrophil, 10^9^/L	1.94 ± 0.01	1.35 ± 0.02	1.66 ± 0.02	2.02 ± 0.02	2.67 ± 0.02	**<.001**
Lymphocyte, 10^9^/L	4.33 ± 0.03	4.31 ± 0.07	4.14 ± 0.06	4.25 ± 0.06	4.62 ± 0.06	**<.001**
Cr, μmol/L	83.17 ± 0.60	93.30 ± 1.82	81.18 ± 0.98	80.59 ± 0.77	78.69 ± 1.02	**<.001**
UA, μmol/L	325.36 ± 1.59	335.62 ± 3.37	318.63 ± 3.04	328.13 ± 3.24	320.06 ± 3.27	**.001**
TG, mmol/L	1.54 ± 0.02	1.42 ± 0.04	1.49 ± 0.03	1.57 ± 0.04	1.68 ± 0.04	**<.001**
TC, mmol/L	5.11 ± 0.03	4.85 ± 0.04	5.14 ± 0.06	5.14 ± 0.04	5.30 ± 0.04	**<.001**
Albumin, g/L	42.00 ± 0.06	38.60 ± 0.11	41.52 ± 0.08	42.88 ± 0.09	44.63 ± 0.11	**<.001**
*Chronic diseases*						
Cardiovascular disease						
Yes	1085 (24.9)	374 (27.9)	277 (19.6)	244 (16.8)	190 (15.6)	**<.001**
No	3281 (75.1)	809 (72.1)	825 (80.4)	858 (83.2)	789 (84.4)	
Diabetes						
Yes	1128 (25.8)	332 (25.9)	291 (21.6)	258 (18.2)	247 (19.8)	**.008**
No	3238 (74.2)	851 (74.1)	811 (78.4)	844 (81.8)	732 (80.2)	
Chronic kidney disease						
Yes	1496 (34.3)	539 (40.5)	358 (25.7)	354 (24.6)	245 (19.0)	**<.001**
No	2870 (65.7)	644 (59.5)	744 (74.3)	748 (75.4)	734 (81.0)	
Hypertension						
Yes	2791 (63.9)	815 (65.5)	700 (58.4)	689 (55.5)	587 (52.8)	**<.001**
No	1574 (36.1)	367 (34.5)	402 (41.6)	413 (44.5)	392 (47.2)	
Cancer category						
Head and neck cancers	187 (4.3)	46 (4.3)	51 (4.8)	45 (4.1)	45 (3.9)	**<.001**
Skin‐related cancers	1289 (29.5)	280 (28.6)	337 (37.4)	358 (41.7)	314 (40.0)	
Respiratory system cancers	102 (2.3)	45 (3.3)	19 (1.5)	17 (1.0)	21 (1.8)	
Digestive system Cancers	361 (8.3)	127 (8.6)	87 (5.5)	87 (5.6)	60 (4.5)	
Breast cancers	635 (14.5)	192 (18.4)	169 (16.3)	154 (14.6)	120 (10.3)	
Blood‐related cancers	77 (1.8)	20 (2.5)	18 (1.9)	19 (2.0)	20 (2.0)	
Musculoskeletal cancers	34 (0.8)	13 (1.0)	4 (0.4)	11 (1.0)	6 (0.7)	
Genitourinary cancers	1449 (33.2)	393 (27.9)	368 (27.7)	360 (25.5)	328 (29.3)	
Other cancers	232 (5.3)	67 (5.4)	49 (4.6)	51 (4.4)	65 (7.5)	

*Note*: Continuous variables were expressed as weighted mean ± standard deviation; one‐way ANOVA was used to compare differences among the different groups. Categorical variables were expressed as weighted frequencies and percentages and compared using Rao‐Scott's *χ* ^2^ test.

Bold values indicate statistical significance (*p* < 0.05).

Abbreviations: Cr, creatinine; UA, serum uric acid; PNI, prognostic nutritional index; TC, total cholesterol; TG, triglyceride.

### 3.2 Association between PNI and ACM, CAM, MTM in American adult cancer patients

With a median follow‐up period of 84 months, a total of 1530 out of 2836 patients (35.04%), 331 out of 2836 (10.45%), and 449 out of 2836 (13.67%) died from ACM, CAM, and MTM, respectively. In this study, we established four multifactorial Cox regression models to examine the relationship between the PNI and ACM, CAM, and MTM among American cancer survivors. Multivariate Cox regression analyses showed a 5.3%, 7.1%, and 5.8% probability reduction in the risk of ACM, CAM, and MTM, respectively, for each 1‐point increase in PNI. After adjusting for various factors, including age, gender, race/ethnicity, marital status, smoking status, BMI, and alcohol consumption, the hazard ratios (HR) were 0.95 (95% CI: 0.93–0.96, *p* < .001) for all‐cause mortality, 0.93 (95% CI: 0.90–0.96, *p* < .001) for cardiovascular mortality, and 0.94 (95% CI: 0.91–0.97, *p* < .001) for malignant tumor mortality in Table 2.

**TABLE 2:** Relationship between PNI and all‐cause mortality cardiovascular mortality, and malignant tumor mortality among adult cancer patients in the United States.

Characteristics	Crude model		Model 1		Model 2		Model 3	
	HR (95% CI)	*p*‐value	HR (95% CI)	*p*‐value	HR (95% CI)	*p*‐value	HR (95% CI)	*p*‐value
All‐cause mortality								
PNI	0.91 (0.89,0.92)	<.001	0.95 (0.93,0.96)	<.001	0.95 (0.94,0.96)	<.001	0.95 (0.94,0.96)	<.001
PNI quartiles								
Q1 (<48.0)	Reference		Reference		Reference		Reference	
Q2 (48.0 to 51.0)	0.465 (0.39,0.55)	<.001	0.617 (0.54,0.71)	<.001	0.63 (0.55,0.73)	<.001	0.65 (0.56,0.76)	<.001
Q3 (51.1 to 54.5)	0.41 (0.34,0.48)	<.001	0.64 (0.55,0.75)	<.001	0.65 (0.55,0.76)	<.001	0.65 (0.55,0.77)	<.001
Q4 (>54.5)	0.29 (0.24,0.35)	<.001	0.52 (0.45,0.62)	<.001	0.521 (0.44,0.62)	<.001	0.52 (0.44,0.62)	<.001
*p* for trend		<.001		<.001		<.001		<.001
Cardiovascular mortality								
PNI	0.873 (0.84,0.90)	<.001	0.929 (0.90,0.96)	<.001	0.93 (0.91,0.96)	<.001	0.93 (0.91,0.96)	<.001
PNI quartiles								
Q1 (<48.0)	Reference		Reference		Reference		Reference	
Q2 (48.0 to 51.0)	0.45 (0.34,0.60)	<.001	0.65 (0.51,0.81)	<.001	0.63 (0.50,0.80)	<.001	0.70 (0.53,0.92)	.01
Q3 (51.1 to 54.5)	0.29 (0.20,0.42)	<.001	0.58 (0.40,0.84)	.004	0.57 (0.39,0.84)	.005	0.61 (0.41,0.89)	.012
Q4 (>54.5)	0.21 (0.13,0.31)	<.001	0.45 (0.32,0.64)	<.001	0.43 (0.31,0.60)	<.001	0.44 (0.31,0.61)	<.001
*p* for trend		<.001		<.001		<.001		<.001
Malignant tumor mortality								
PNI	0.908 (0.881,0.936)	<.001	0.942 (0.913,0.971)	<.001	0.940 (0.911,0.971)	<.001	0.943 (0.913,0.973)	<.001
PNI quartiles								
Q1 (<48.0)	Reference		Reference		Reference		Reference	
Q2 (48.0 to 51.0)	0.31 (0.23,0.43)	<.001	0.40 (0.29,0.54)	<.001	0.39 (0.29,0.54)	<.001	0.41 (0.30,0.56)	<.001
Q3 (51.1 to 54.5)	0.42 (0.32,0.55)	<.001	0.62 (0.47,0.81)	<.001	0.61 (0.47,0.80)	<.001	0.62 (0.47,0.82)	<.001
Q4 (>54.5)	0.26 (0.19,0.36)	<.001	0.42 (0.31,0.56)	<.001	0.40 (0.29,0.54)	<.001	0.41 (0.30,0.56)	<.001
*p* for trend		<.001		<.001		<.001		<.001

*Note*: Calculated using multivariate COX regression analysis was performed, weight. Crude Model: no adjustment. Model 1: adjusted for age, sex, marital status, race/ethnicity, BMI, smoking status, and alcohol use. Model 2: adjusted for age, sex, marital status, race/ethnicity, BMI, smoking status, alcohol use, neutrophil count, creatinine, uric acid, triglycerides, and total cholesterol. Model 3: adjusted for age, sex, marital status, race/ethnicity, BMI, smoking status, alcohol use, neutrophil count, creatinine, uric acid, triglycerides, total cholesterol, hypertension, diabetes, cardiovascular disease, and chronic kidney disease.

Meanwhile, after further additional adjustments for laboratory biochemical parameters, the correlation between PNI and the risk of all‐cause mortality, cardiovascular mortality, and death from malignancy remained a strong (HR: 0.95, [95% CI: 0.94–0.96], *p* < .001; HR = 0.93, [95% CI: 0.91–0.96], *p* < .001; HR = 0.94, [95% CI: 0.91–0.97], *p* < .001). Each unit increase in PNI was associated with a reduction probability of 5.1%, 6.7%, and 6.0% in the risk of death in cancer patients. Furthermore, even after adjusting for potential confounding variables (Model 3), PNI significantly reduced the risk of ACM, CAM, and MTM in cancer patients (HR: 0.95, [95% CI: 0.93–0.94], *p* < .001; HR: 0.93, [95% CI: 0.91–0.96], *p* < .001; HR: 0.94, [95% CI: 0.91–0.97], *p* < .001), as shown in Table 2.

**FIGURE 2:**
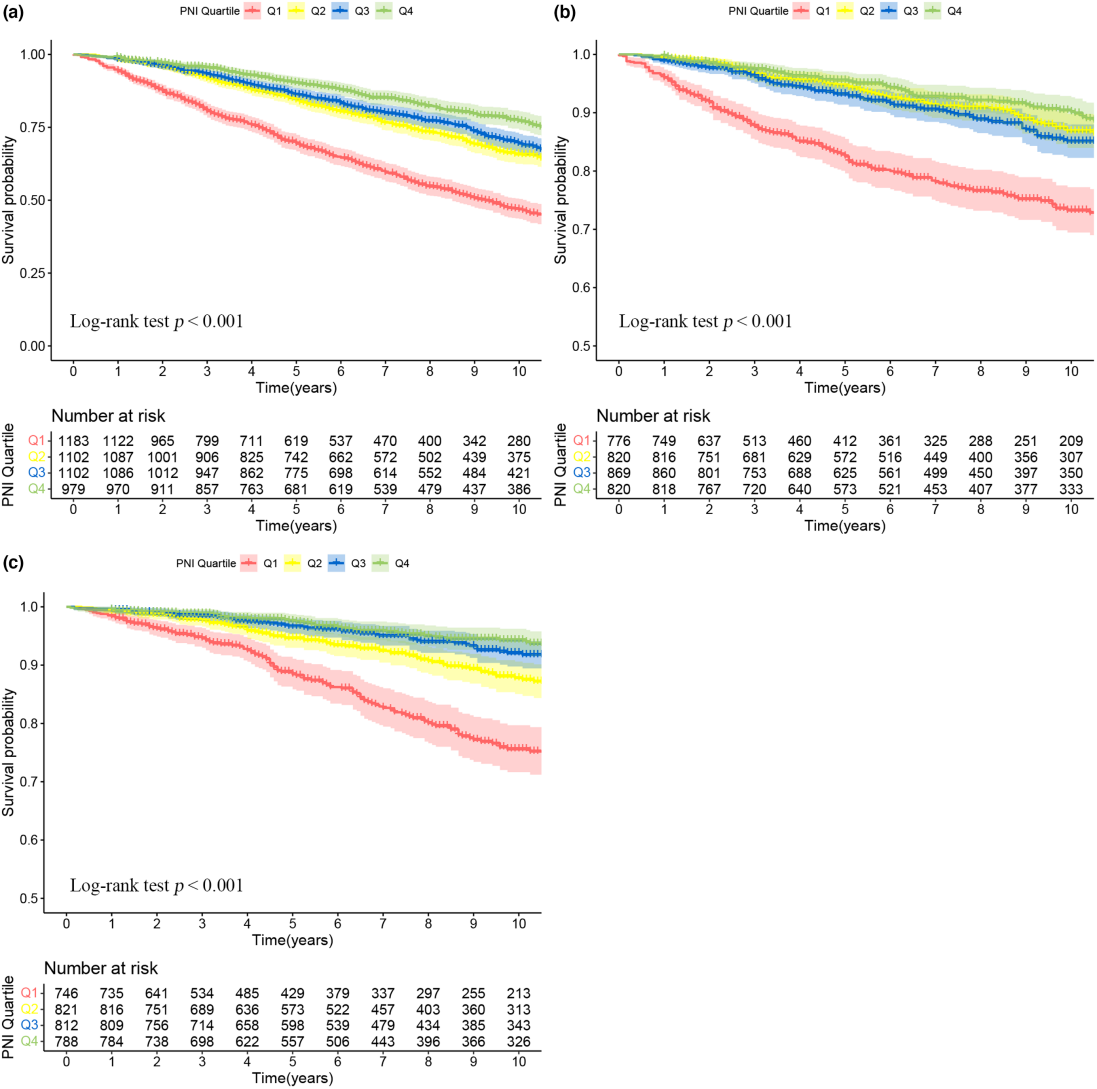
Kaplan–Meier survival rates illustrating mortality among US adult cancer patients categorized by different PNI quartile groups: (a) all‐cause mortality; (b) cardiovascular mortality; (c) malignant tumor mortality. PNI stratification: Q1 (<48.0), Q2 (48.0 to 51.0), Q3 (51.1 to 54.5), and Q4 (>54.5). Statistical adjustments were made for age, gender, marital status, ethnicity, BMI, smoking status, alcohol use, neutrophil count, creatinine, uric acid, triglycerides, total cholesterol, diabetes, hypertension, chronic kidney disease, and cardiovascular disease.

In addition, Figure 2 shows Kaplan–Meier survival curves for ACM, CAM, and MTM of survival in American adult cancer patients stratified by PNI group (Figure 2a–c). The curves depicted those individuals with higher PNIs had lower mortality rates compared to those with lower PNIs, with a significant difference (log‐rank *p* < .001).

### 3.3 Nonlinear correlation analysis of PNI and ACM, CAM, MTM in American adult cancer patients

**FIGURE 3:**
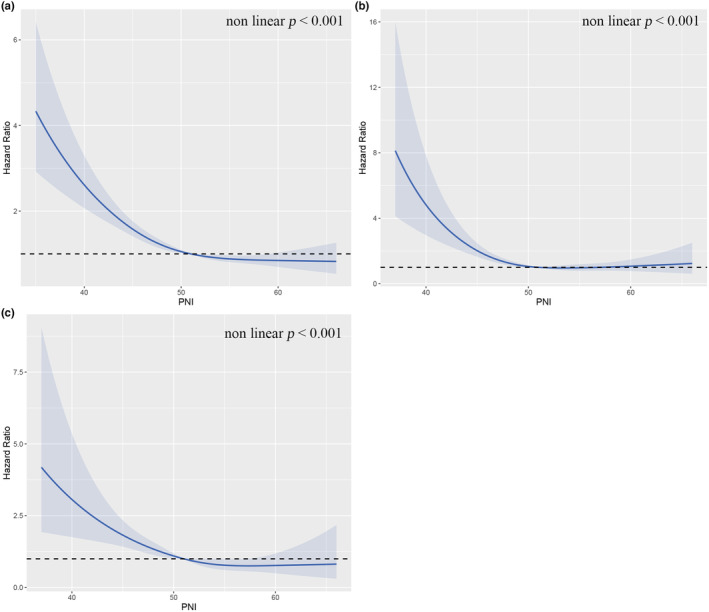
Nonlinear relationship between PNI and mortality in adult cancer patients. (a) all‐cause mortality; (b) cardiovascular mortality; (c) malignant tumor mortality. Statistical adjustments were made for age, gender, race/ethnicity, marital status, smoking status, BMI, alcohol consumption, neutrophil count, triglycerides, serum creatinine, total cholesterol, serum uric acid, diabetes, hypertension, chronic kidney disease, and cardiovascular disease.

In the restricted cubic spline plot (Figure 3), the horizontal axis represents the percentage of PNI, while the vertical axis indicates the HR of ACM, CAM, and MTM incidence. The results revealed a nonlinear correlation between PNI and the risk of ACM, CAM, and MTM (nonlinear *p* < .001). Furthermore, there was an “L”‐shaped curve correlation between PNI and the occurrence of all‐cause deaths, cardiovascular deaths, and deaths from malignant tumors.

### 3.4 Subgroup analysis of PNI and ACM, CAM, MTM in American adult cancer patients

We further analyzed subgroups by gender, age, and Cancer Category. The results suggest that regardless of age (≤ 60 or >60) or gender (male or female), an increase in PNI was associated with a significant reduction in the risk of ACM among US adult cancer patients. At the same time, PNI showed a protective effect against ACM in different categories of malignancies, except for blood‐related cancers. Furthermore, we observed a protective effect of PNI on the risk of CAM and MTM in participants aged over 60 years, as well as in both male and female participants. However, this association was not observed in patients aged ≤60 years (Table 3).

**TABLE 3:** Associations between PNI and all‐cause mortality cardiovascular mortality, and malignant tumor mortality in different subgroups of American Adult Cancer Patients.

Characteristics	All‐cause mortality		Malignant tumor mortality		Cardiovascular mortality	
	HR (95% CI) *p*	*p* for interaction	HR (95% CI) *p*	*p* for interaction	HR (95% CI) *p*	*p* for interaction
Age						
<60	0.94 (0.90,0.99) ***p* = .012**	.885	0.94 (0.87,1.02) *p* = .125	.806	0.90 (0.74, 1.06) *p* = .196	.703
≥60	0.94 (0.92,0.95) ***p* < .001**		0.93 (0.90,0.97) ***p* < .001**		0.91 (0.89,0.94) ***p* < .001**	
Gender						
Male	0.93 (0.91,0.95) ***p* < .001**	.571	0.92 (0.87,0.97) ***p* < .001**	.186	0.87 (0.84,0.91) ***p* < .001**	.14
Female	0.94 (0.92,0.96) ***p* < .001**		0.95 (0.91,0.99) ***p* = .007**		0.93 (0.88, 0.98) ***p* = .005**	
Race						
Non‐Hispanic White	0.93 (0.92,0.94) ***p* < .001**	.077	0.93 (0.90,0.96) ***p* < .001**	.637	0.90 (0.87,0.93) ***p* < .001**	.958
Non‐Hispanic Black	0.96 (0.93,1.00) ***p* = .040**		0.95 (0.89, 1.02) *p* = .151		0.91 (0.82, 1.02) *p* = .098	
Mexican American	0.94 (0.87, 1.02) *p* = .125		0.92 (0.81, 1.04) *p* = .183		0.86 (0.63, 1.18) *p* = .360	
Other race	0.88 (0.82,0.94) ***p* < .001**		0.86 (0.75, 0.98) ***p* = .019**		0.85 (0.62,1.17) *p* = .325	
Cancer category						
Head and neck cancers	0.89 (0.82, 0.97) ***p* = .009**	**.016**	0.90(0.79, 1.03) *p* = .133	.228	1.09 (1.01,1.18) ***p* = .037**	.12
Skin‐related cancers	0.93 (0.90,0.96) ***p* < .001**		0.96 (0.88, 1.05) *p* = .386		0.87 (0.82,0.93) ***p* < .001**	
Respiratory system cancers	0.91 (0.87, 0.95) ***p* < .001**		0.83 (0.74,0.93) ***p* = .002**		0.93 (0.85, 1.02) *p* = .119	
Digestive system cancers	0.94 (0.91,0.98) ***p* = .004**		0.93 (0.87, 1.01) ***p* = .075**		0.90 (0.83,0.97) ***p* = .009**	
Breast cancers	0.95 (0.91,0.99) ***p* = .016**		0.94 (0.87, 1.02) *p* = .131		0.91 (0.81, 1.02) *p* = .116	
Blood‐related cancers	0.94 (0.84, 1.04) *p* = .229		0.95 (0.80, 1.12) *p* = .527		3.45 (3.11,3.82) ***p* < .001**	
Musculoskeletal cancers	0.61 (0.43, 0.88) ***p* = .008**		0.70 (0.51,0.95) ***p* = .022**		7.46 (0.41,1.37) *p* = .344	
Genitourinary cancers	0.94 (0.92,0.96) ***p* < .001**		0.94 (0.90,0.99) ***p* = .021**		0.90 (0.86,0.95) ***p* < .001**	
Other cancers	0.87 (0.82,0.92) ***p* < .001**		0.89 (0.81, 0.98) ***p* = .012**		0.84 (0.76, 0.93) ***p* < .001**	

*Note*: Calculated using multivariate COX regression analysis was performed, weight. Adjusted for age, sex, marital status, race/ethnicity, BMI, smoking status, alcohol use, neutrophil count, creatinine, uric acid, triglycerides, total cholesterol, hypertension, diabetes, cardiovascular disease, and chronic kidney disease.

Bold values indicate statistical significance (*p* < 0.05).

Furthermore, we did find that PNI significantly attenuated cardiovascular deaths in US adult cancer patients with increasing PNI in the strata of respiratory system cancers, musculoskeletal cancers, genitourinary cancers, and other cancers. Similarly, PNI significantly reduced the incidence of malignancy deaths in US adult cancer patients with increasing PNI in the subgroups of skin‐related cancers, digestive system cancers, genitourinary cancers, and other cancers. However, there was a positive correlation between PNI and malignancy deaths in the subgroups of head and neck cancers and blood‐related cancers.

## 4 DISCUSSION

Through the retrospective analysis of a large cohort of adult cancer survivors in America, the study reveals that the PNI is a potentially independent risk factor for prognosis. The study simultaneously explored the relationship between PNI and ACM, CAM, and MTM in cancer survivors separately. The findings show an L‐shaped relationship between PNI and adverse outcomes in cancer survivors, indicating that patients with lower PNI scores have a higher probability of death, regardless of ACM, CAM, or MTM. The results of the subgroup analyses showed a negative association between PNI and the risk of ACM among adult cancer patients in the United States, except for blood‐related cancers. However, it should be noted that the association between PNI and the risk of CAM and MTM showed inconsistent results across subgroups. Furthermore, it is interesting that the PNI demonstrated good predictability for ACM, CAM, and MTM in non‐Hispanic white (*p* < .05).

The study sample size is larger compared to previous studies, and it is the first study conducted in a nationally representative adult cancer population. Currently, the study of the relationship between nutritional and inflammatory indicators and tumor prognosis has become an important topic. Some studies (Mirili et al., 2019; Nazha et al., 2015) have suggested that nutrition and inflammation play a vital role in tumor development, angiogenesis, and apoptosis. This finding is in accordance with previous studies (Ellez et al., 2023; Kocak et al., 2023; Maejima et al., 2022; Xu et al., 2023) and supports the usefulness of PNI as a predictor of prognosis in cancer patients. However, the differing results in subgroup analyses may be attributed to variations in the stratified population, race, and genetic inheritance, among other factors.

The PNI, which is calculated from serum albumin levels and total lymphocyte counts, serves as a predictor of nutritional status. It has been widely used not only to predict poor prognosis in a variety of patients but also has been considered a significant predictor of cancer (Lu et al., 2021; Obermair et al., 2017; Yoshihisa et al., 2018). The PNI is an objective measure as it is derived from routine laboratory indicators that can be easily obtained in a clinical setting. Clinical caregivers can use the PNI to assess a patient's condition quickly, enabling timely interventions to reduce the risk of death. Moreover, it allows for large‐sample monitoring and follow‐up. Therefore, improving PNI scores by controlling inflammation and enhancing immune status may prove to be an effective clinical intervention tool.

The mechanisms underlying the association between lower PNI scores and worse survival in cancer patients are not yet well understood, because albumin and lymphocytes are two important factors in the PNI equation, which may explain the relationship between PNI and mortality in cancer survivors. According to the former study (Lu et al., 2021), it demonstrated the relationship between PNI and a patient's prognosis that low serum albumin often suggests malnutrition in the patient. Not only does serum albumin reflect the body's nutritional status, which regulates the body's inflammatory response and has a strong negative correlation with C‐reactive protein (CRP), but influences the function of many oxidative stress enzymes in the body (Deng et al., 2021; Eckart et al., 2020; Li et al., 2023). Furthermore, the lymphocyte reflects not only the body's immune regulation but also the progression of inflammation and its role as a fundamental component of cytotoxic immune function, mediating cytotoxicity through cytokines, thereby inhibiting tumor cell proliferation and invasion (Ray‐Coquard et al., 2009; Walzik et al., 2021). Currently, it has been demonstrated that controlling inflammation and improving immunity can improve the prognosis of patients (Shimizu et al., 2022; Zhang, Feng, et al., 2023). Similarly, a significant reduction in cardiovascular risk in mice has been found to influence the expression of inflammatory cell‐associated factors and chemokines (Wang et al., 2022). Therefore, specific mechanisms can be considered in terms of immune function and inflammatory response when exploring the relationship between lower PNI and worse prognosis in cancer patients.

Interestingly, however, we also found that PNI was associated with cancer‐related mortality and did not apply to blood‐related cancers. Blood‐related cancers, such as leukemia and myeloma, possess distinct different pathophysiological characteristics than other types of solid tumors. These cancers occur predominantly in the bone marrow or lymphoid tissue and do not form masses like solid tumors. As a result, we believe that the effect of PNI on blood‐related cancers may be minimal or insignificant. Second, there may still be other important factors that influence mortality from blood‐related cancers. For instance, patients with this cancer are treated differently and have a different prognosis than those with solid tumors. It is essential to carefully consider the specific type of cancer when interpreting the association between PNI and cancer‐related mortality. PNI may not be the primary influencing factor for blood‐related cancers, while other factors might exert a more significant influence. Nevertheless, there is no doubt that the relationship between PNI and blood‐related tumors needs to be further investigated.

Several limitations were observed in the study. For example, since the data only encompassed US adult cancer patients, the findings may not be extrapolated to participants in other countries with differing genetic backgrounds, diets, and lifestyles. In addition, despite our attempts to adjust for numerous confounding factors, the potential omission of other significant covariates remains plausible.

## 5 CONCLUSION

The PNI score emerges as an independent risk factor for ACM, CAM, and MTM from malignant tumors in cancer survivors, with higher PNI scores associated with lower mortality. Therefore, assessing cancer patients' immune and nutritional statuses via PNI proves helpful in lowering poor prognosis risks and is crucial for comprehensive cancer patient management.

## AUTHOR CONTRIBUTIONS

**Li Zhao:** Data curation (equal); formal analysis (equal); methodology (equal); resources (equal); software (equal); writing – original draft (equal). **Xia Shen:** Data curation (equal); formal analysis (equal); investigation (equal); methodology (equal); software (equal); writing – original draft (equal). **Long Yang:** Investigation (equal); validation (equal); writing – review and editing (equal). **Pengfei Wang:** Formal analysis (equal); investigation (equal); writing – review and editing (equal). **Jianfeng Zhang:** Data curation (equal); investigation (equal); writing – review and editing (equal). **Ning Liu:** Supervision (equal). **Yan Xie:** Supervision (equal).

## FUNDING INFORMATION

External funding was not received for this study.

## CONFLICT OF INTEREST STATEMENT

The authors state that there are no conflicts of interest.

## ETHICS STATEMENT

NHANES obtained ethical approval from the Ethics Review Committee of the National Center for Health Statistics, under the following protocol numbers: #2018‐01, Continuation of Protocol #2011‐17, Protocol #2011‐17, Continuation of Protocol #2005‐06, Protocol #2005‐06, and Protocol #98‐12. You can access the pertinent details at: https://www.cdc.gov/nchs/nhanes/irba98.htm. As this study constituted a secondary analysis of NHANES data, obtaining patient informed consent was not required.

## PATIENT CONSENT STATEMENT

This study involves an analysis of publicly available NHANES data. Informed consent was originally obtained from NHANES participants by the National Center for Health Statistics Research Ethics Review Board.

## Data Availability

All the data are publicly accessible and can be obtained from the website: https://wwwn.cdc.gov/nchs/nhanes/search/default.aspx.
